# Analysis of Cyclin D1 in Breast Cancer: A Call to Arms

**DOI:** 10.1007/s12609-012-0083-7

**Published:** 2012-06-22

**Authors:** Nicholas P. Tobin, Jonas Bergh

**Affiliations:** 1 Cancer Center Karolinska, Karolinska Institutet and University Hospital, 171 76 Stockholm, Sweden; 2 Manchester University, Manchester, M20 4BX England UK

**Keywords:** Cyclin D1, Breast cancer, Amplification, Protein expression, Independent analysis, Molecular subgroups, Ki67

## Abstract

The oncogenic capabilities of the cell cycle protein cyclin D1 have long been established in a breast cancer setting. The *CCND1* gene is amplified in up to 15 % of breast tumors, with overexpression of its corresponding protein found in up to 50 % of cases. While gene amplification is consistently associated with reduced patient survival times and treatment resistance, repeated attempts to clarify the prognostic and predictive impact of the cyclin D1 protein in breast cancer have yielded contrasting results. Here, we recommend that any examination of cyclin D1 in a patient cohort should begin by determining *CCND1* copy number, with subsequent removal and separate analysis of amplified cases. Next, the remaining tumors should be examined for cyclin D1 protein expression in the context of well-defined breast cancer subgroups. Only in this manner can the true clinical value of cyclin D1 be fully elucidated.

## Background

The cyclin family of cell cycle regulatory proteins coalesces with their binding partners, the cyclin-dependent kinases (CDKs), to strictly govern the incremental steps leading to mammalian cell division. Consecutive expression of cyclins D, E, A, and B are essential for the successful transition of a cell through the G1, S, and G2/M phases of cell cycle, respectively. As such, perturbations in these proteins, resulting in uncontrolled proliferation, can be considered an oncogenic event. The 34 kDa G1 to S-phase transition marker cyclin D1 represents the best studied cell cycle protein and has been well established as a human oncogene in a breast cancer setting. Upon complex formation with CDK 4/6, cyclin D1 phosphorylates and inactivates the retinoblastoma (Rb) protein, resulting in the release of E2F transcription factors and progression of cell cycle. While the protein’s biological role would appear straightforward, studies examining the relationship between cyclin D1 and clinical outcome of breast cancer patients have yielded inconsistent findings. Here, we explore the potential shortcomings of how cyclin D1 is currently analyzed and recommend improvements, with the aim of enhancing its clinical utility.

## The Need to Resolve Conflicting Results

Cyclin D1 has remained a putative prognostic and predictive biomarker in breast cancer for well over a decade. However, repeated clinical analyses producing conflicting results have left this promising marker teetering on the edge of consignment to the “never quite made it” file. This would be a harsh injustice, for if we take a step back and delve into the information we have garnered thus far, it is readily apparent that its potential has been limited only by our misinterpretation of cyclin D1 amplification and protein expression data. To be more precise, we have failed to consistently separate these two very different biological phenomena when determining the relevance of cyclin D1 in a breast cancer setting.

Amplification of the cyclin D1 gene (*CCND1*) occurs in 15 % of primary estrogen receptor (ER) positive breast cancers, while overexpression of the protein is found in 50 % of cases. These figures are well established and indicate that mechanisms other than gene amplification are responsible for dysregulation of the protein. The vast majority of breast tumors bearing *CCND1* amplification are readily defined as ER positive, are luminal B by gene expression analysis, and overexpress cyclin D1 protein; most notably, patients with *CCND1* amplified tumors show reduced survival times and associations to treatment resistance. Remarkably, this picture of dynamic clinical utility in both a prognostic and predictive setting has been blurred by conflicting assessments of the relationship between cyclin D1 protein levels and clinicopathological parameters.

Overexpression of cyclin D1 protein has been linked with both *positive* (most likely due to associations with ER + tumors) and *negative* breast cancer prognosis. However, the relevancy of these findings is often ambiguous, owing to antibody disparities and low patient numbers, resulting in underpowered conclusions. Further confusing matters, tumors high in cyclin D1 protein have been linked with resistance to endocrine therapy and shorter recurrence-free survival of breast cancer patients. These uncertain results directly contrast the consistent message provided by *CCND1* amplified tumors. This highlights the necessity of separating patients with amplification of the gene for independent analysis *before* relating expression of cyclin D1 protein to clinicopathological data. Given that the vast majority of *CCND1* amplified tumors overexpress cyclin D1 protein and have poor prognosis, they represent a separate entity and should be treated as such. In any analysis examining the relevance of cyclin D1 protein in breast cancer, failing to remove the *CCND1* amplified cases biases the cyclin D1 overexpressed group by artificially inflating the number of cyclin D1 “high” tumors with a worse clinical outcome. The significance of this should not be underestimated; it is not common practice to remove amplified cases before conducting cyclin D1 protein analysis. Thus, we cannot say with any certainty how the protein influences patient survival and response to treatment.

Proceeding in this manner will allow us to determine the protein’s true relationship to breast cancer outcome and could even alter how we interpret its expression. For instance, instead of thinking of cyclin D1 as a cell cycle marker, it could be thought of as an indicator for an intact and functional ER. When bound to estrogen, ER upregulates cyclin D1 mRNA and protein expression, resulting in normal to elevated levels of cyclin D1 protein in ER + tumors. Additionally, cyclin D1 has the ability to upregulate ER in the absence of estrogen. This evidence implies that low expression of the protein in ER + tumors could be interpreted as a loss of control over ER signaling and as an oncogenic indicator. However, this concept is somewhat obscured by the ability of pathways including MAP kinase to stimulate cyclin D1 transcription independently of ER. Nevertheless, the fact remains that tumors associated with ER positivity, specifically those in the luminal breast cancer subgroups, tend to express more cyclin D1 on the mRNA and protein levels than do those that are ER negative.

Similar pursuits for functions of cyclin D1 outside of its classical cell cycle role have led to the elucidation of its associations to epithelial-to-mesenchymal transition, to cell invasion/migration, and as a promoter of Notch1 expression. These studies not only highlight why we should refrain from casting cyclin D1 as a mere cell cycle regulator, but also provide evidence of the protein’s complex interactions. Indeed, given these diverse capabilities, it should come as little surprise that such conflicting results have been observed when its protein levels have been related to patient outcome. This makes the argument for analyzing gene amplified cases separately all the more relevant, and furthermore, if the biological interplay of the protein is so diverse, dividing tumors into clinically relevant subgroups should form an intrinsic part of any analysis.

## Subgroup Analysis of Cyclin D1 Protein Expression

Tumor pathology and gene expression profiling has taught us to view breast cancer as a complex, heterogeneous disease, but one that can be separated into biologically relevant subgroups using the clinical biomarkers ER, progesterone receptor, human epidermal growth factor receptor 2 (HER2), and Ki67. In addition to differences in underlying biology, patients in these subgroups also display diverging responses to tumor therapy, sites of tumor relapse, and survival time. Given these broad differences, it is unlikely that cyclin D1 protein has the same clinical relevance in all breast cancer subtypes. Consider the case of a luminal B breast tumor: This subgroup is strongly ER positive and displays elevated levels of cyclin D1 protein. In contrast, the basal subgroup of breast cancer is typified by its ER negativity and low cyclin D1 expression. On the basis of this, low expression of cyclin D1 in a luminal tumor would represent a deviation from the normal and potentially would indicate disrupted ER signaling; but in a basal tumor, the opposite is true, and high expression would be considered an aberration. Since most breast cancer cohorts contain approximately 70 % luminal tumors, the relevance of cyclin D1 in smaller subgroups such as basal tumors is lost, demonstrating why subgroup analysis is so important.

Finally, an alternate strategy in the analysis of cyclin D1 could be to group tumors on the basis of how the protein relates to a cell proliferation, using a marker such as Ki67. In its fundamental role, cyclin D1 indicates progression of the cell cycle and acts as a sign that a cell population is actively proliferating. However, its cyclic nature prevents its use as a bona fide proliferation marker, a task better suited to the routinely employed Ki67 protein. Notably, cyclin D1 has been both positively and negatively correlated to Ki67 expression, raising an interesting question worthy of exploratory analysis: Can the relationship between Ki67 and cyclin D1 predict clinical outcome and potentially indicate treatment resistance? For example, if cyclin D1 protein and Ki67 are both elevated in a tumor, does this denote an intact proliferative cycle and a better response to therapy? This relationship is rarely considered in cyclin D1 protein analysis and certainly never after having removed *CCND1* amplified cases from the equation first.

## Summary and Recommendations

Clearly, if cyclin D1 is to succeed as a prognostic or treatment predictive biomarker in breast cancer, a stronger selection criterion must be imposed on tumor samples to achieve consistent results. If researchers persist in analyzing cyclin D1 protein expression without first removing *CCND1* amplified cases, we will continue to see conflicting, variable results, much to the demise of this promising biomarker. We therefore recommend that any examination of cyclin D1 in a clinical material must begin with a determination of *CCND1* copy number and subsequent independent analysis of any amplified cases. Next, protein expression of the remaining samples should be analyzed in the context of clinically relevant subgroups by separating tumors based on ER, PR, HER2, and Ki67 expression. While this may result in few cases in each subgroup, it is preferable to the alternative of trying to apply a single biomarker to range of diverse, biologically distinct tumors. If these strategies are rigorously applied, they will solidify the importance of *CCND1* amplification as a clinical marker and will allow us to determine the relevance of aberrant cyclin D1 protein expression in breast tumors.

